# Thermodilution-Based Invasive Assessment of Absolute Coronary Blood Flow and Microvascular Resistance: Quantification of Microvascular (Dys)Function?

**DOI:** 10.1155/2020/5024971

**Published:** 2020-11-17

**Authors:** Daniëlle C. J. Keulards, Mohamed El Farissi, Pim. A. L Tonino, Koen Teeuwen, Pieter-Jan Vlaar, Eduard van Hagen, Inge. F. Wijnbergen, Annemiek de Vos, Guus R. G. Brueren, Marcel van't Veer, Nico H. J. Pijls

**Affiliations:** ^1^Catharina Hospital, Eindhoven, Netherlands; ^2^Eindhoven University of Technology, 5612 AZ Eindhoven, Netherlands

## Abstract

During the last two decades, there has been a sharp increase in both interest and knowledge about the coronary microcirculation. Since these small vessels are not visible by the human eye, physiologic measurements should be used to characterize their function. The invasive methods presently used (coronary flow reserve (CFR) and index of microvascular resistance (IMR)) are operator-dependent and mandate the use of adenosine to induce hyperemia. In recent years, a new thermodilution-based method for measurement of absolute coronary blood flow and microvascular resistance has been proposed and initial procedural problems have been overcome. Presently, the technique is easy to perform using the Rayflow infusion catheter and the Coroventis software. The method is accurate, reproducible, and completely operator-independent. This method has been validated noninvasively against the current golden standard for flow assessment: Positron Emission Tomography-Computed Tomography (PET-CT). In addition, absolute flow and resistance measurements have proved to be safe, both periprocedurally and at long-term follow-up. With an increasing number of studies being performed, this method has great potential for better understanding and quantification of microvascular disease.

## 1. Invasive Diagnosis of Microvascular Disease: Time for a Leap Forward?

In the last two decades, there has been a sharp increase in publications about the coronary microcirculation with more than 200 new articles per year. It reflects a new interest for the microcirculation. It is fully accepted nowadays that epicardial coronary artery disease is not the only pathologic entity in ischemic heart disease and that the microcirculation has been underappreciated for years. It has been recognized that 25–50% of patients with chest pain visiting the catherization laboratory do not present with significant epicardial stenosis: so-called Angina with Nonobstructive Coronary Artery (ANOCA) disease or Myocardial Infarction with Nonobstructive Coronary Artery (MINOCA) disease [1]. Moreover, a considerable number of patients continue to have chest complaints even after successful percutaneous coronary intervention (PCI) of epicardial lesions [2]. Due to this mismatch between patients' symptoms and epicardial angiography, assessment of the coronary microcirculation or microvasculature has gained interest [3].

Coronary microvascular disease (CMVD) can be divided into roughly three categories: (1) CMVD in the absence of obstructive coronary artery disease (primary CMVD), (2) CMVD secondary to myocardial diseases, for example, left ventricular hypertrophy and Takotsubo, and (3) CMVD in the presence of obstructive coronary artery disease [4]. Most likely, primary microvascular dysfunction is caused by a combination of factors being intimal thickening, smooth muscle cell proliferation, and molecular mechanisms [4]. The microcirculation is too small to be depicted by traditional invasive imaging methods. Therefore, only functional methods can be used to evaluate the microcirculation in the catheterization laboratory. There are several invasive and noninvasive methods focusing on microcirculatory pressure and flow. This review discusses a new and promising invasive method for easy and accurate assessment of absolute coronary blood flow and microvascular resistance using thermodilution and low rate infusion of saline.

## 2. When to Assess Microvascular Function and/or Coronary Vasospasm?

Due to the increased interest and recognition of microvascular coronary artery disease and coronary vasospasm, more of such patients present at the outpatient clinic or emergency ward. The question that remains is who should undergo further invasive analysis and which investigational methods can be used.

The diagnosis ANOCA, as mentioned, is used to characterize patients with chest pain but with normal/near-normal coronary arteries. Many different pathologies can cause ANOCA as previously mentioned. The first suspicion for the physician follows from the anamnesis and/or cardiac enzymes or functional testing. It is important to realize that patients presenting with “chest pain” are not always comparable and that patients with ANOCA often present with slightly different symptoms, that is, chest tightness after exercise or at rest, dyspnea, and so forth [5].

Some patients present with a myocardial infarction and marked elevated cardiac enzymes without coronary stenosis (MINOCA). Most of these patients with obvious chest/dyspnea complaints undergo some sort of additional noninvasive diagnostic test like bike-treadmill testing or coronary CT-angiography. When no abnormalities are found but clinical suspicion remains, an invasive coronary angiogram can be planned. In the cath lab, the endothelium-dependent and endothelium-independent causes of ANOCA can be distinguished. The current EAPCI consensus document on INOCA states that vasospastic coronary artery disease could, and sometimes should, be assessed within the invasive angiography session (class IIa recommendation) [5]. Coronary vasospasm is also called endothelium-dependent microvascular disease and can be assessed after the administration of increasing doses of intracoronary acetylcholine (ACH). ACH normally binds to the ACH-receptor and causes vasodilatation in the endothelial cell. ACH always causes slight simultaneous constriction of the vascular smooth muscle cell, but in healthy endothelium the net product is vasodilatation [6]. In case of abnormal endothelial cells, ACH binds to the ACH-receptor and does not cause vasodilatation and the slight simultaneous constriction of the smooth muscle cells leading to a net constriction. During ACH testing, a standard approach involves sequential infusion of ACH at concentrations approximating 10^−6^, 10^−5^, and 10^−4^ mol/L. Epicardial spasm can be diagnosed when the epicardial coronary artery is narrowed >90% after administration of the ACH, accompanied by recognizable complaints and ECG changes corresponding to ischemia. The full protocol is also presented in the consensus document mentioned above [5].

**Figure 1:**
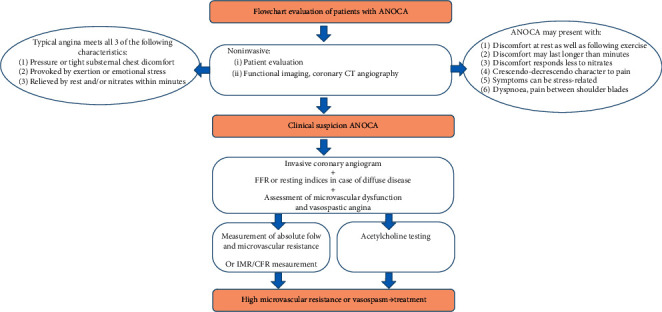
The evaluation of ANOCA patients in a flowchart. ANOCA: Angina with Nonobstructive Coronary Arteries, CT: computed tomography, FFR: fractional flow reserve, IMR: index of microvascular resistance, and CFR: coronary flow reserve.

Existing high microvascular resistance is called endothelium-independent microvascular disease. Maximal vasodilatory hyperemia is always caused by the intracoronary (or intravenous) administration of endothelium-independent vasodilators, that is, saline in the case of absolute flow and resistance measurement using the dedicated Rayflow catheter (but also adenosine/regadenoson when measuring IMR/CFR). The full description of the measurement method is explained in the chapter below. IMR and CFR measurement has already been explained extensively previously [7] and goes beyond the scope of this article. The overall diagnostic path that can be followed for ANOCA is displayed in Figure 1.

## 3. Measurement of Absolute Blood Flow and Resistance

**Figure 2:**
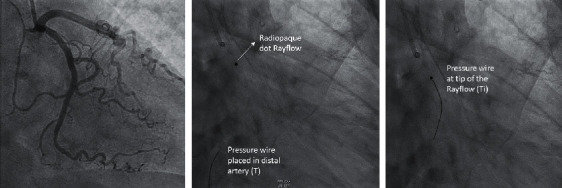
Pressure wire and infusion catheter position. In the left panel, a normal circumflex artery is shown. The middle panel shows the pressure wire X, which is placed in the distal coronary artery, and the Rayflow catheter in the proximal artery. The Rayflow is visible by a radiopaque dot at the tip. In this position, the measurement starts. The right panel shows the position of the pressure wire when it is pulled back towards the inner side holes of the Rayflow catheter. Now the infusion temperature is assessed. *T*_*i*_: the infusion temperature of the saline as measured at the infusion holes of the Rayflow catheter; *T*: the distal coronary temperature after complete mixing of blood and saline measured by the pressure wire after calibration to body temperature.

To apply this technique, cardiac catheterization and/or FFR can be performed according to routine by either femoral or radial access. Guiding catheters should be advanced as usual and next a pressure/temperature wire (Pressure wire X™ Abbott, Saint Paul, MN, USA) is introduced in the ostium of the coronary artery. After intracoronary administration of 200 micrograms of nitroglycerin and proper equalization of pressures, the pressure wire can be further advanced into the coronary artery in addition to, if desired, assessment of epicardial abnormality using FFR or nonhyperemic pressure ratios (NHPR). Fractional flow reserve (FFR) is measured by intravenous administration of adenosine; RFR (available using the Coroventis software) does not require adenosine and is preferred in some centers. Following epicardial assessment, a dedicated monorail infusion catheter (Rayflow™, Hexacath, Paris) is advanced over the pressure wire and positioned with its tip in the proximal part of the coronary artery (Figure 2).

**Figure 3:**
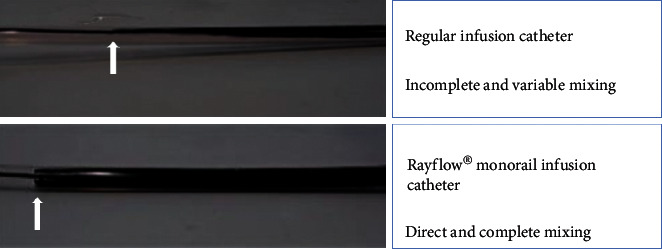
Difference in infusion between normal catheter and Rayflow. In the upper panel, an in vitro setup of a coronary artery is shown. The regular infusion catheter is placed in the “vessel” filled with saline and the infusate is dyed black with ink. It is visible that there is incomplete and variable mixing in case of the regular infusion catheter (arrow indicates tip of infusion catheter). In the lower panel, the Rayflow is used. Here immediate and complete mixing is shown, starting directly at the infusion holes (indicated by the arrow).

**Figure 4:**
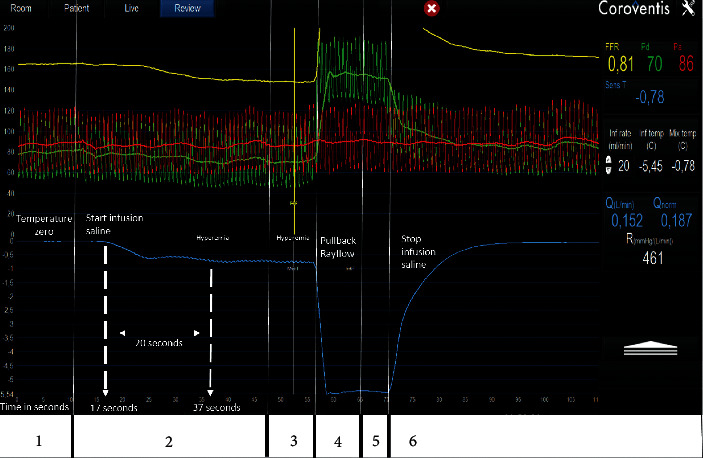
Measurement screen, step-by-step. This figure shows the software screen during the flow and resistance measurement. Panel 1: the temperature measured by the pressure wire is zeroed, which means it is calibrated at body temperature. Panel 2: the infusion of saline starts at 20 ml/min in this case; the fast decrease in temperature is visible here. Panel 3: steady-state maximum hyperemia has been reached here and *T* is recorded. Panel 4: the pressure wire is pulled back to the tip of the Rayflow to measure the infusion temperature of the saline. This pullback is indicated by the fast decrease in temperature and sudden increase in distal pressure. Panel 5: the infusion temperature measurement reaches steady state and *T*_*i*_ is calculated. Panel 6: the infusion pump is stopped and the temperature of the blood reaches starting values within 30 seconds. Further, the timeline in the figure indicates the time in seconds. Here, it can be appreciated that it takes approximately 20 seconds for hyperemia to occur. *Red tracing: aortic pressure; green tracing: distal coronary pressure; blue tracing: coronary temperature. The numerical values of all relevant parameters are displayed in the right side of the Coroventis screen*. *T*_*i*_: the infusion temperature of the saline as measured at the infusion holes of the Rayflow catheter; *T*: the distal coronary temperature after complete mixing of blood and saline measured by the pressure wire after calibration to body temperature.

This infusion catheter has an outer diameter of 0.84 mm (2.5 French) and it consists of a 25 cm long rapid exchange inner monorail lumen for the 0.014″ pressure wire and an infusion lumen along the complete length of the catheter. The catheter is equipped with 4 infusion holes at a distance of 7 mm from its tip mandatory for rapid and complete mixing of saline with blood in the coronary artery (Figure 3). In addition, the infusion catheter has two inner side holes approximately 1 cm from the tip between the infusion lumen and the monorail lumen to record precisely the temperature of saline at the spot where it enters the coronary artery. Before saline infusion starts, the temperature is calibrated and body temperature is set to “zero” (reference temperature, Figure 4, panel 1), where after all changes in temperature are related to this reference temperature. During the measurement, the sensor of the pressure/temperature wire is positioned in the distal part of the coronary artery.

Next, saline infusion is started at a rate of 15–25 ml/min (*Q*_*i*_) and absolute blood flow in the coronary artery is calculated as previously described [8–10]. Maximum hyperemia in the respective coronary artery is induced by the saline infusion itself within 10–20 seconds [11, 12].

During steady-state infusion, the temperature of the completely mixed blood and saline (*T*) is measured in the distal coronary artery after a steady state has been reached (Figure 4, panel 3); the pressure wire is pulled back in the Rayflow catheter to determine the infusion temperature of the saline (*T*_*i*_) (Figure 4, panel 4). Absolute blood flow is then calculated by the following equation:

(1) $$\begin{array}{c} Q_{b}=1.08\frac{T_{i}}{T}Q_{i}, \end{array}$$

where *Q*_*b*_ is the hyperemic coronary blood flow in ml/min. *T*_*i*_ is the infusion temperature of the saline as measured at the infusion holes of the Rayflow catheter. *T* is the distal coronary temperature after complete mixing of blood and saline measured by the pressure wire. Both *T* and *T*_*i*_ are measured as a difference to body temperature (calibrated to 0). *Q*_*i*_ is the infusion rate of saline in ml/min. The constant value 1.08 relates to the difference between the specific heats and densities of blood and saline.

Because also distal coronary pressure (*Pd*) is recorded simultaneously, the microvascular resistance (*R*) can be calculated in analogy to Ohm's law by dividing the distal pressure and flow by the following simplified equation:

(2) $$\begin{array}{c} R=\frac{Pd}{Qb}. \end{array}$$

**Figure 5:**
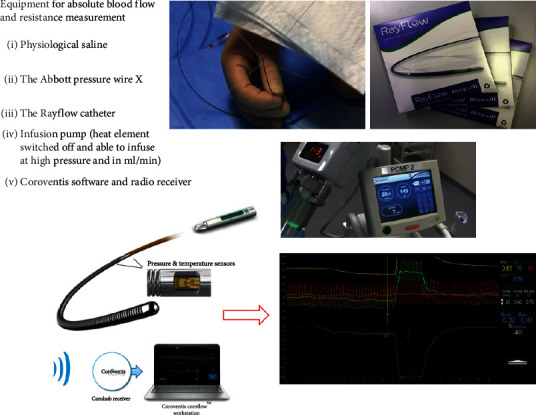
Equipment. All equipment needed for the thermodilution-based assessment of absolute blood flow and resistance is displayed here. The Rayflow catheter is shown in the upper panel, clearly indicating the 4 infusion holes at 0°, 90°, 180°, and 270°. The middle panel shows the infusion pump. The lower panel shows the pressure wire with the necessary software.

All signals are instantaneously displayed on the regular cath lab monitor by dedicated software (Coroflow®, Coroventis, Uppsala, Sweden; Figures 4 and 5). This software displays not only all pressure parameters and fractional flow reserve but also absolute blood flow, the normal value of absolute blood flow (obtained by *Q*_*b*_/FFR), and microvascular resistance (in mmHg/L/min or WU).

## 4. Advantages Compared to Present Methods

The first and most important advantage of the absolute flow and resistance measurement is the ability to measure absolute blood flow and microvascular resistance truly quantitatively. CFR reflects the ratio between basal and hyperemic coronary flows. This tells us something about both the epicardial and microcirculatory functions lumped together but does not enable distinguishing between these two compartments. CFR can be estimated by a ratio of flow velocities, measured by Doppler. CFR can also be approximated by thermodilution during an IMR measurement using a bolus of saline to measure mean transit time (Tmn) at rest and during maximum hyperemia. CFR_thermo_ is represented then by the ratio of mean transit times [7]. Use of CFR in clinical practice is limited because of its variability due to blood pressure, heart rate, vessel diameter, age, and others. This causes a large variation in “normal” values and values can vary significantly within the same patient over time. In addition, Doppler techniques are sensitive to slight motion of the patients, breathing, and minimal position changes of the sensor and as such are operator-dependent. IMR itself is not influenced by blood pressure, heart rate, or vessel diameter but is still operator-dependent due to the injection technique of boluses of saline. IMR software tries to limit variability by taking the mean of three measurements. In contrast, the absolute flow and resistance measurements are completely operator-independent: once the infusion of saline has started, the operator stands back, while steady-state measurements are performed. Measurements can be repeated quickly if desired.

Also, by infusing saline though the dedicated Rayflow catheter, maximum hyperemia occurs within 10–20 seconds after start of infusion and no additional hyperemic stimulus is necessary as tested extensively by De Bruyne et al. [11–13]. The absence of need for adenosine makes this method very patient-friendly, since hyperemia by the saline infusion itself is not causing chest discomfort in the vast majority of patients. In a large safety study in only 2% of the patients, mild chest discomfort was noticed [8, 14]. This is in contrast to the frequently observed (although innocent) chest pain observed during hyperemia induced by intravenous adenosine [15, 16]. Therefore, although generally 30–60 seconds is sufficient to perform the flow and resistance measurements, these measurements can be safely continued for minutes if desired and without any side effect.

The safety of absolute flow and resistance method has been investigated, both periprocedurally and at long-term follow-up [14]. Except for short rapid transient conduction disturbances in 2.6% of measurements, no noticeable side effects were observed periprocedurally and at follow-up of 30 days and 1 year; no adverse event or index vessel related revascularization could be attributed to these measurements.

Reproducibility is excellent [8] and the complete measurement procedure takes only a few minutes in addition to FFR or RFR measurement. As such, a complete evaluation of both the epicardial and the microvascular compartments can be performed easily and quickly. Lastly, the method was validated against the current golden standard for flow assessment: the PET-CT [13].

## 5. Short Overview of the Necessary Equipment

In contrast to IMR, to perform the measurement as explained above, some specific equipment is needed in addition to the pressure wire. First, as a matter of fact the Rayflow multipurpose monorail infusion catheter is mandatory to infuse the saline and to guarantee complete mixing of blood and saline, a prerequisite for reliable measurement (Figures 3 and 5). Second, a programmable infusion pump is used, which should be able to infuse saline at a rate of 15–25 ml/min at high pressure. Finally, for rapid and instantaneous recording of all relevant parameters and the calculations made, presence of Coroflow software and a radio receiver (Coroflow®, Coroventis, Uppsala, Sweden) is mandatory. All equipment is summarized in Figure 5.

## 6. Practical Issues

Like with every invasive imaging modality, there are some practical issues to keep in mind when using this technique. First, the Rayflow catheter is compatible with a 6F guiding and 6F introducer sheath. Next, the heating element of the infusion pump should be switched off. Next, it is to make sure there is enough saline in the infusion pump to complete the whole measurement. For one measurement, generally 50 cc of saline is needed. Further it is important to wait long enough till maximum hyperemia occurs and all values (*Pa*, *Pd*, and *T*) stabilize before pulling back the pressure wire into the Rayflow catheter for measurement of *T*_*i*_. Such steady state is generally achieved within 20–30 seconds and this ensures correct values and high reproducibility.

In the event of an AV-block (sometimes seen in small RCA when using an infusion rate above 15 ml/min), the infusion pump should be stopped and AV-conduction recovers immediately. After several seconds, the pump can be adjusted to a lower infusion rate and the measurement can be repeated. All cases of AV-block in the aforementioned safety study disappeared immediately after stopping the infusion and medication was never required to recover AV-conduction [14]. Finally, it should be kept in mind that calculated flow refers to maximum flow distal to the tip of the Rayflow catheter and that resistance refers to minimal resistance of the myocardium corresponding to that position.

## 7. Studies Performed Previously, Limitations, and Future Applications

As a matter of fact, patients with ANOCA, MINOCA, syndrome X, mismatch between epicardial abnormalities and chest pain, and a multitude of primary myocardial diseases will be the focus for quantifying microvascular function. In that regard, and for any interindividual comparison, need for normal values is obvious. In a large recently performed study, ranges of normal values were defined and were as expected quite large due to dependency of resistance on the mass of the myocardial territory distal to the spot of measurement [17].

To exclude the extent of the myocardial territory as a variable, it is recommendable to express flow per gram of tissue (ml/min/g) and resistance as resistance × gram of tissue (WU × g). In that case, mass should be obtained from coronary CT and MRI. That has been done recently and when doing so, circumscribed narrow ranges of normal minimal resistance were obtained, equal for all 3 major perfusion territories [18].

For intraindividual follow-up of microvascular disease and effects of treatment, this methodology is extremely suitable because every patient or myocardial territory has its own control. A number of studies have been performed or are presently performed in this respect. Already years ago, when equipment was less refined, Wijnbergen et al. [19] studied changes in myocardial resistance from directly after STEMI PPCI to days to weeks of follow-up. It was suggested that a normal or increased value of resistance measurement after PPCI, which restored considerably at follow-up, was associated with favorable outcome, whereas a persistent high resistance would be unfavorable [19]. Larger studies are mandatory to relate such measurements to outcome, in analogy to IMR studies [7, 20]. This method was also recently used to better understand the recovery of the microcirculation after PCI of chronic occlusions [21, 22]. Here flow and resistance measurements were performed directly after CTO PCI and at follow-up. Recovery of both absolute blood flow and myocardial resistance was observed over time.

Currently there are a couple of interesting trials using this technique. The ongoing IMPACT-CTO 2 trial (ClinicalTrials.gov Identifier: NCT03830853) combines absolute flow and resistance assessment, FFR, RFR, and IMR with intracoronary imaging within the same patient after CTO PCI and at long-term follow-up. These measurements might provide further insight into coronary physiology and anatomy after CTO PCI. Another study to be mentioned in this context is the prospective multicenter randomized placebo-controlled EDIT-CMD (EudraCT number: 2018‐003518‐41) study, where patients with chest pain of uncertain origin, microcirculation, and effects of calcium antagonists, are studied before treatment and after 6 weeks of calcium-antagonist treatment.

Finally, in case of attempted pharmacologic treatment or risk factor modification of microvascular disease, the measurements described in this paper may prove a suitable instrument for recording progression or regression of disease. Larger studies are required to draw more definite conclusions, especially in different clinical scenarios, without limiting ourselves to ANOCA/MINOCA. With the wide field of applications of this harmless technique, the studies mentioned are only the tip of the iceberg. Multiple areas of further research exist.

## 8. Absolute Flow and Resistance Measurement: the Future Invasive Standard for the Coronary Microcirculation?

Taking into account its ease of use, safety, accuracy, reproducibility, and the capability for specific and quantitative characterization of the coronary microvasculature, the measurement of absolute coronary blood flow and microvascular resistance can be proposed as the future standard for invasive assessment of microvascular (dys)function.

## Abbreviations

ANOCA: Angina with Nonobstructive Coronary Arteries
CAG: Coronary angiogram
CFR: Coronary flow reserve
CMVD: Coronary microvascular disease
FFR: Fractional flow reserve
IMR: Index of microvascular resistance (*U*)
LAD: Left anterior descending
LCx: Ramus circumflex
MINOCA: Myocardial Infarction with Nonobstructive Coronary Arteries
PCI: Percutaneous coronary intervention
*Pd*: Distal pressure (mmHg)
*Q*: Flow (ml/min)
*R*: Resistance (wood units)
RCA: Right coronary artery
*T*: Distal coronary temperature (°C)
*T*_*i*_: Infusion temperature (°C)
WU: Wood units (mmHg/L/min).
